# Determinants of Telehealth Continuance Intention: A Multi-Perspective Framework

**DOI:** 10.3390/healthcare10102038

**Published:** 2022-10-15

**Authors:** Hui-Lung Hsieh, Jhih-Ming Lai, Bi-Kun Chuang, Chung-Hung Tsai

**Affiliations:** 1 Chu Shang Show Chwan Hospital, Nantou 557, Taiwan; 2 Department of Information Technology and Management, Tzu Chi University of Science and Technology, Hualien 973302, Taiwan

**Keywords:** the theory of planned behavior, technology acceptance model, self-determination theory, telehealth

## Abstract

Owing to the COVID-19 pandemic, many countries’ physicians in the world have used telehealth to visit patients via telehealth. The study aimed to integrate the theory of planned behavior (TPB), the technology acceptance model (TAM), and self-determination theory (SDT) to explore the adoption behavior of a telehealth system. A convenient sample of residents was drawn from the population of Nantou County in Taiwan and analyzed via structural equation modeling. The findings revealed that attitude, perceived behavioral control, perceived usefulness, and perceived autonomy support jointly have significantly positive effects on continuance intention. Results also confirmed that perceived autonomy support, perceived ease of use, and perceived usefulness jointly have significantly positive effects on attitude. Furthermore, this study also showed that a crucial mediators’ role is played by perceived ease of use, perceived usefulness, and attitude. The conclusions and practical implications of the research will hopefully provide health organizations and institutions with some innovative insights and foresights, which in turn will promote better practices and services of telehealth technology.

## 1. Introduction

In late 2019, the SARS-CoV-2 virus caused the COVID-19 global pandemic, bringing a major impact on people’s lives. Under the pandemic, home isolation or quarantine has become one of the most important methods to avoid the risk of infection. In addition, countries around the world have implemented lockdown policies to stop the spread of the virus. Isolation, quarantine and lockdown have posed great challenges to the normal face-to-face approach to medical care. To ensure access to medical care, the Ministry of Health and Welfare of Taiwan issued a letter in February 2020 stating that healthcare providers may follow the Rules of Medical Diagnosis and Treatment by Telecommunications to extend telemedicine services, so that local health bureaus and designated medical institutions may also conduct telemedicine [1]. Prior to the COVID-19 pandemic, only 20% of primary care physicians in the U.S. and 16% in Canada interacted with patients using video consultations [2]. Due to the pandemic, telehealth has been rapidly gaining attention and adoption around the world as an innovative way to deliver health care services. Studies have also shown that telehealth has significant potential to enhance health care practice [3] and to reduce the spread of the virus [4], especially during the COVID-19 pandemic.

In recent years, as the concept of health promotion and preventive medicine has become more popular, health behaviors and self-care management have become more important to the public. Therefore, the study of health behavior models will be important in designing effective interventions to improve self-health management [5]. The Theory of Planned Behavior (TPB) is a health behavior model that is widely used in the health care field. Many studies have shown that the TPB has good explanatory power for exploring health behaviors. In addition, many studies in recent years have applied TPB to the field of telemedicine and telehealth. At the same time, many studies have also extensively used the Technology Acceptance Model (TAM) to investigate the intentions and behaviors of medical, nursing, and long-term care-related information technology use. TAM is also a suitable tool for explaining the use behavior of telehealth, which is a popular topic today [6]. In addition, another theory that focuses on people’s motivation is Self-Determination Theory (SDT), which considers motivation as the psychological energy that moves people toward a particular goal. Many theories explain the orientation of people’s behavior but fail to explain the reinforcement of people’s behavior. SDT emphasizes the influence of the role of social context on motivation and behavior, and thus offers a new approach to the study of health behavior [7]. All three of these theories have been widely applied to the field of health behavior, thus demonstrating that the empirical results generally support the hypothetical arguments. However, a more comprehensive and complete research approach that integrates the three theories would be beneficial for the future development of telehealth.

Accordingly, this study attempts to integrate the Theory of Planned Behavior, the Technology Acceptance Model, and Self-Determination Theory to construct a cross-theoretical research framework focusing on the behaviors of people in the community who use telehealth. It is hoped that this innovative care technology can further evolve during the COVID-19 pandemic.

## 2. Literature Review

### 2.1. Telehealth and COVID-19

Telehealth involves information and communication technologies, such as telephone and video conferencing, for synchronous or asynchronous consultations to overcome geographic or functional distances [2]. Telehealth allows patients to meet with their care providers online to monitor their health status remotely or to receive information about specific diseases, enabling the delivery of health care services across geographic distances [8]. Broadly speaking, there are two types of telehealth systems, synchronous and asynchronous. Synchronous systems allow healthcare professionals to see or consult with patients/clients online in real time, while asynchronous systems store the electronic medical records of patients/clients on the local end and then send that information to a call center or medical facility on the remote end. Both methods allow the health care providers to conduct contact, diagnosis, and treatment without the patient/client having to be in the same location [9]. Telehealth applies technologies such as video conferencing, the internet, and store-and-forward systems. The demonstrated benefits of telehealth include improved continuity of care, accessibility, and health outcomes [10]. According to Gajarawala and Pelkowski [4], the benefits of telehealth include: providing access to resources and care for patients in remote or underserved areas, improving efficiency without increasing costs, reducing patients’ travel and waiting times, improving quality of care, and increasing patient satisfaction.

The COVID-19 pandemic has posed a severe limitation for in-person health care, making bigger barriers than ever to health care accessibility [11]. In the wake of the COVID-19 outbreak, lockdown policies around the world in response to the spread of the virus have led to the use of digital technology to achieve virtual healthcare and minimize in-person contacts. Telehealth can significantly reduce in-person contacts in healthcare, with benefits such as preventing shortages of personal protective equipment, reducing nosocomial infections, protecting health care workers from infection, and rebuilding clinical practice for medical students [12]. Telehealth adopts information and communication technologies to provide health care services for the purpose of enhancing health care, public health, and health education. In a severe pandemic, telehealth provides access to these critical supports and services, enabling communities and families to access high-effective interventions that enhance the well-being of the population [13]. In Taiwan, in response to the rising pandemic situation, the Ministry of Health and Welfare (MOHW) announced on 15 May 2021 that it would promote the establishment of video clinics in designated hospitals nationwide and would also open up the possibility of telephone consultation for revisiting patients whose conditions are assessed to be stable by doctors, until the end of the month following the downgrading or lifting of the national Level 3 alert. In addition, on 22 December 2020, the National Health Insurance Administration announced the “National Health Insurance Telemedicine Benefit Plan”, which includes telemedicine in mountainous and outlying areas into national health insurance coverage. It can be seen that due to the aging population in Taiwan, the demand for health management services is increasing, and the COVID-19 pandemic has accelerated the implementation and popularization of telehealth, making this innovative medical service even more prosperous.

### 2.2. Theory of Planned Behavior (TPB)

TPB is derived from the Theory of Reasoned Action (TRA), which has been widely applied to predict people’s specific behaviors. According to TRA, an individual performing a given behavior depends on his behavioral intention. A person’s attitude and subjective norm for this behavior together determine behavioral intention. Behavioral intention is a person’s readiness to perform a given behavior. Attitude is an assessment of what a person favors or does not favor about engaging in the behavior. They are more likely to engage in the behavior if they believe it is beneficial to them. Subjective norm, on the other hand, is social pressure to perform or not perform the behavior and refers to the perceptions of important others regarding the given behavior [14,15,16,17]. To address the limitation that people may not have full voluntary control over the behavior, Ajzen [18] extended TRA to include perceived behavioral control which then became TPB. Perceived behavioral control is a person’s perception of their ability to engage in the behavior. If they believe that the behavior is attainable, they are more likely to perform the behavior. This concept reflects the internal (confidence in the ability to engage in the behavior) or external (availability of resources to engage in the behavior) constraints on engaging in the behavior [15,16,17,19].

In social psychology, TPB has become one of the most influential theories in the studies of intentional behavior and has been widely used to predict and understand people’s health behaviors [20]. Related studies include: binge drinking [21], physical activity [22,23], fruit and vegetable intake, breakfast intake, snack avoidance [24], exercise [25], and healthy eating [26]. In the area of telehealth, there are also many studies using TPB to examine users’ adoption behaviors. Kuo et al. [27] used TPB to explore the adoption behaviors of 15 hospital physicians in Taiwan and found that attitude, subjective norm, perceived behavioral control, and behavioral intention were positively associated. AlBar and Hoque [28] conducted a study on patients using e-health in Saudi Arabia and found that attitude and subjective norm significantly influenced patients’ behavioral intentions. Winkelmann et al. [29] used the TPB questionnaire with a study of athletic trainers and orthopedic physicians and found that those who had adopted telehealth were more consistent with all concepts of TPB than those who had not. Ramírez-Correa et al. [17] used an online questionnaire to study the acceptability of telehealth among Brazilian adults during the COVID-19 pandemic. The study found that TPB provided more significant explanatory power than TAM.

In summary, the theoretical hypothesis remains valid in general, based on past adoption of TPB in studies related to telehealth. Therefore, we propose the following hypotheses.

**H1:**  *Attitude positively affects continuance intention in a telehealth context.*

**H2:**  *Subjective norm positively affects continuance intention in a telehealth context.*

**H3:**  *Perceived behavioral control positively affects continuance intention in a telehealth context.*

### 2.3. Technology Acceptance Model (TAM)

The Technology Acceptance Model (TAM) has developed into a theory with great influence and value in explaining information technology (IT) use behavior [30,31]. Based on the Theory of Reasoned Action (TRA) [14], TAM asserts that perceived usefulness and perceived ease of use are the main relevant factors for IT acceptance behavior. Perceived usefulness refers to a user’s subjective perception of how using a particular IT will improve their task performance. Perceived ease of use, on the other hand, refers to the degree to which users expect the IT to be easy to use. Perceived usefulness and perceived ease of use are two beliefs that influence intention to use through attitude (the user’s attitude toward using the given technology). Perceived usefulness directly affects attitude, while perceived ease of use directly affects perceived usefulness. Use behavior depends on the intention to use. Moreover, perceived usefulness and perceived ease of use are affected by external factors [32]. Many subsequent empirical studies have found that TAM can consistently account for a significant proportion (approximately 40%) of variance in use intention and behavior [33]. Among the studies, Venkatesh and Davis [33] integrated TAM with social influence and cognitive instrumental processes to form TAM2. The social influence process variables include: subjective norm, voluntariness, and image. The cognitive instrumental process includes: job relevance, output quality, and result demonstrability. Venkatesh et al. [34] further integrated the eight user acceptance models into an integrated theory of technology use and acceptance, which pointed out that performance expectancy, effort expectancy, social influence, and facilitating conditions are the main factors which directly affect the intention and behavior of users.

In the health care industry, professionals are highly educated, highly trained with medical expertise who work in a high stress environment. Due to the complexity of the industry and its unique occupational variability, adapting TAM to fit the context of the health care field is another direction of development [31]. Even in the case of telehealth, TAM is also an appropriate tool for explaining use behaviors [6]. Or et al. [15] used TAM to examine the acceptance behaviors of home care patients with an interactive website as the subject of their study. The study found that perceived usefulness significantly influenced behavioral intention. Kohnke et al. [35] took patients with heart diseases, chronic obstructive pulmonary diseases (COPDs), diabetes, and hypertension and physicians who had used telehealth as research subjects and concluded that perceived usefulness and perceived ease of use significantly influenced intention to use. Tsai [36] conducted a survey on people who used telehealth from a remote area of Taiwan, integrating the Social Capital Theory, the Social Cognitive Theory, and the Technology Acceptance Model as the theoretical model, with empirical results also supporting the TAM hypotheses. Bettiga et al. [6] focused on preventive health care and explored the use of smart mobile health care systems, finding that perceived usefulness and perceived ease of use significantly influenced intention to use and further influenced willingness to pay. Peixoto et al. [3] studied patients’ acceptance of teleconsultation in Brazil during the COVID-19 pandemic. The results of the study indicated that the TAM hypotheses held except for the effect of perceived ease of use on attitude, which was not significant.

In summary, the theoretical hypothesis remains valid in general, based on past adoption of TAM in studies related to telehealth. Therefore, we propose the following hypotheses.

**H4:**  *Perceived usefulness positively affects continuance intention in a telehealth context.*

**H5:**  *Perceived usefulness positively affects attitude in a telehealth context.*

**H6:**  *Perceived ease of use positively affects attitude in a telehealth context.*

**H7:**  *Perceived ease of use positively affects perceived usefulness in a telehealth context.*

### 2.4. Self-Determination Theory (SDT) and Perceived Autonomy Support

Aside from TPB, another recent social psychology theory that has been widely applied to health behavior is the Self-Determination Theory (SDT). SDT suggests that people’s intrinsic growth tendencies and innate psychological needs: competence, relatedness, and autonomy are the basis for intrinsic motivation. SDT classifies people’s motivation into three forms: autonomous, controlled, and amotivation. Autonomous is the most self-determined form of motivation, where a person engages in a behavior based on the intrinsic value of the behavior or to achieve an important personal value or goal. Such behaviors come from the individual’s true feelings and is therefore called self-determination. Conversely, if one engages in behavior based on external pressures or obligations, the motivation is controlled. Amotivation, on the other hand, is the lack of motivation to act, where one either does not perform the action or does not act intentionally [37,38,39]. Among the three types of motivation, having autonomous motivation increases the participation and continuity of behavior. With controlled motivation, a person will stop the behavior once the external command or control disappears [38,40]. Autonomous motivation reflects that a person engages in a behavior because it satisfies personal goals and fulfills three innate psychological needs: autonomy, competence, and relatedness [41].

SDT is a theory that examines the effect of social factors on intention, where a measure of non-stressful forms of social influence is proposed. Self-determination theory posits that social contexts have a pervasive influence on an individual’s motivation and psychological well-being [22]. Therefore, establishing a perceived autonomy support context or environment is an effective way to promote autonomous motivation and behavioral change [40]. Perceived autonomy support is defined as the beliefs of people that significant others support the individual’s self-initiation, opportunities for choice, independent problem solving, involvement in decision making, acknowledgement of feelings, and avoiding making pressurizing commands [41]. A context of perceived autonomy support is one that adopts the individual’s perspectives, encourages and responds to their problems, supports their initiatives, provides choices, and reduces control [39]. A social environment for full autonomy support includes: (1) choice, e.g., significant others encourage choice and participate in decision making; (2) rationale, e.g., significant others explain in a meaningful way how the performance of an action is important; (3) acknowledgment, e.g., significant others minimize stress and acknowledge the individual’s feelings and perspective [22,23,24,42]. Conversely, when people are in social contexts that are controlling or not fully autonomy-supportive, they experience environments that force or coerce them to act in particular ways [23]. In summary, autonomous motivation can be nurtured in a social environment of perceived autonomy support provided by significant others, which in turn facilitates behavioral engagement and continuity. Because motivation is an important factor in health behaviors, some research has applied SDT’s perspective and theoretical framework to study the adoption of information systems [43,44] and telehealth [45].

### 2.5. The Integration of SDT and TPB

Many studies have suggested that the integration of motivational factors into TPB would be useful for the interpretation of health behaviors. This integrative approach suggests that motivational and social contextual factors from SDT are distal predictors of the TPB model’s variables, and that the TPB model’s variables are the proximal influences on behavior. This theory is based on the tenet of SDT that individuals should integrate their beliefs and motivations for future behaviors in order to engage in behaviors. Therefore, an environment and motivation that promotes autonomous behavior will contribute to the strengthening of positive beliefs and the formation of behavioral intentions [22,23,40,46,47]. Evidence that perceived autonomy support promotes autonomous motivation, which in turn influences health behaviors, has been supported by numerous studies in the health care field. Chatzisarantis, Hagger, and Smith [22] found in a study of physical activity that perceived autonomy support directly and indirectly predicted behavioral intention through attitude. Subsequent studies have similarly demonstrated that perceived autonomy support directly and indirectly predicts behavioral intention through attitude [23]. Moreover, a study on myopia prevention showed that perceived autonomy support significantly influenced autonomous motivation, which in turn influenced attitude, subjective norm, perceived behavioral control, and then reading distance [46]. A study by Girelli et al. [24] on three types of healthy eating behaviors noted that perceived autonomy support was mediated through attitude, subjective norm, and perceived behavioral control, and in turn influenced healthy eating behaviors. A study by Chung et al. [40] on seasonal influenza prevention behaviors showed that perceived autonomy support among staff at a Hong Kong elderly center was positively and significantly associated with autonomous motivation to wear masks and indirectly influenced their intentions to wear masks through mediation of attitudes, subjective norms, and perceived behavioral control. Sicilia et al. [48] who integrated SDT and TPB to study an adolescent population, also showed that perceived autonomy support influences intention to exercise through attitude, subjective norm, and perceived behavioral control.

In summary, perceived autonomy support affects the continuance intention of an information system directly or indirectly through attitude, subjective norm, and perceived behavioral control. Therefore, we propose the following hypotheses.

**H8:**  *Perceived autonomy support positively affects continuance intention in a telehealth context.*

**H9:**  *Perceived autonomy support positively affects attitude in a telehealth context.*

**H10:**  *Perceived autonomy support positively affects subjective norm in a telehealth context.*

**H11:**  *Perceived autonomy support positively affects perceived behavioral control in a telehealth context.*

### 2.6. The Integration of SDT and TAM

Davis et al. [49] introduced motivational factors into the TAM with respect to information system use. Motivation is divided into two types: extrinsic and intrinsic. Extrinsic motivation is defined as engaging in an action because of the perception that it will help achieve a valuable outcome, rather than because of the interest in engaging in the action itself. Intrinsic motivation, on the other hand, is defined as engaging in an action not because of external reinforcement but because of the interest in the action itself. Perceived usefulness is an extrinsic motivation; perceived ease of use has both an extrinsic and intrinsic mechanism of motivation; and perceived enjoyment is an intrinsic motivation. Venkatesh [50] indicated that intrinsic motivation is computer playfulness, which is defined as the degree of cognitive spontaneity in computer interactions. Igbaria et al. [51] integrated perceived ease of use, extrinsic motivation (perceived usefulness) and intrinsic motivation (perceived enjoyment) to investigate the effect on application use. Igbaria et al. [52] proposed a motivational model of microcomputer use and confirmed that extrinsic motivation (perceived usefulness), intrinsic motivation (perceived enjoyment), and social pressure are factors that influence individuals’ decisions to use computers. Teo et al. [53] and Teo [54] also found that ease of use, extrinsic motivation (perceived usefulness), and intrinsic motivation (perceived enjoyment) were key factors in internet use behaviors. Moon and Kim [55] proposed the concept of perceived playfulness, which represents people’s intrinsically important beliefs and explains intrinsically motivating behaviors. Many subsequent researchers in information systems have begun to integrate motivational factors into the TAM model to explore the effects of motivational factors on attitudes, beliefs, and behaviors [56].

The integration of SDT with TAM has been widely used and validated by many studies in recent years. SDT posits that the social contextual conditions that support people’s autonomy, competence, and relatedness are the basis for increasing intrinsic and extrinsic motivation and lead to better performance. Since autonomy-supportive environments increase the propensity for particular behaviors, providing reasons, choices, and encouraging critical thinking are more likely to foster autonomy. Roca & Gagné [56] introduced SDT’s autonomy support, competence, and relatedness to TAM to explore the effect of the three concepts on beliefs (perceived ease of use and perceived usefulness) and intention to use e-learning technology. The findings revealed that perceived autonomy support directly and significantly influenced perceived ease of use, while perceived autonomy support indirectly and significantly influenced perceived ease of use through perceived playfulness. Lee et al. [57] also showed that teachers’ care, assistance, and inclusion (perceived autonomy support) significantly influenced students’ perceived usefulness of e-learning systems. Yuan and Liu [58] conducted a survey on QQ, the most popular messaging software in China, and showed that the autonomy support felt by QQ group members and the experience without external pressure increased their autonomy motivation to share knowledge and thus their willingness to share knowledge. Wang’s study [59] on social software pointed out that users’ perceived autonomy support influences their posting behavior through autonomy motivation. Many recent studies also indicate that the psychological needs of autonomy, competence, and relatedness have significant positive effects on the perceived ease of use and perceived usefulness of IT [60,61,62,63,64]. 

In summary, perceived autonomy support affects the continuance intention to use information systems through perceived ease of use and perceived usefulness. Therefore, we propose the following hypotheses.

**H12:**  *Perceived autonomy support positively affects perceived ease of use in a telehealth context.*

**H13:**  *Perceived autonomy support positively affects perceived usefulness in a telehealth context.*

**Figure 1:**
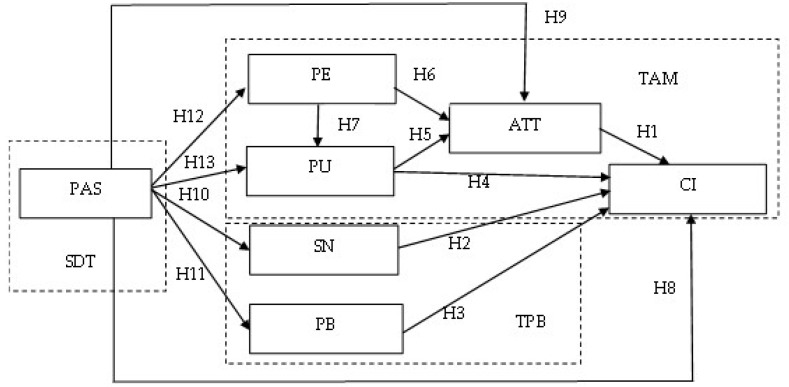
The Proposed Research Model. Note: PAS: Perceived Autonomy Support, PE: Perceived Ease of Use, PU: Perceived Usefulness, SN: Subjective Norm, PB: Perceived Behavior Control, ATT: Attitude, CI: Continuance intention.

As discussed above, we constructed the proposed research model, which is illustrated in Figure 1.

## 3. Method

### 3.1. Measures

In this study, a cross-sectional questionnaire research method was used, and a questionnaire scale was developed to test the hypotheses of the research framework. The questionnaire questions were developed from the literature cited in the previous section, modified to fit the context of telehealth, and developed into a preliminary draft. The questionnaire was divided into demographic variables (gender, age, and education), and measures of each construct (perceived autonomy support, perceived ease of use, perceived usefulness, subjective norm, perceived behavioral control, attitude, and continuance intention). The questions for each construct were measured on a five-point Likert scale, where 1 represents “strongly disagree” and 5 represents “strongly agree”. The questions of the seven constructs were modified from previous research scales. The perceived autonomy support scale was modified from [41], the perceived ease of use and perceived usefulness scale was modified from [65], and the subjective norm, perceived behavioral control, attitude, and continuance intention scale was modified from [19]. 

Scholars and experts in the health care field were invited to review the first draft of the questionnaire for validity, ensuring that the questions were comprehensive, relevant, and representative. Finally, the revised questionnaire was pre-tested with community residents in order to adjust the wording to avoid misunderstandings or ambiguities that might affect the correctness of responses.

### 3.2. Sample

The community-based telehealth system was provided by Chu Shang Show Chwan Hospital and was installed at community care centers across the Nantou County. The system functions of physiological signals measurement included blood pressure, blood glucose, heart rate, etc. The vital signs were measured at remote centers and then transmitted to the health data management platform of the hospital’s call center for immediate monitoring and follow-up health management and medical diagnosis. If there is any abnormality in the physiological data of an elderly individual in the community, a warning message will be displayed on the health data management platform and the duty officer will notify the elderly by phone to schedule a return visit or emergency care.

The subjects of this study were residents in Nantou County, Taiwan, who had used community-based telehealth care services. Subjects were interviewed in person for a questionnaire survey. Subjects filled in the questionnaire themselves based on their subjective feelings, or the interviewers filled in the questionnaire according to the answers given by the subjects. A total of 400 questionnaires were sent out and collected, and the information obtained was collated. After deducting the invalid questionnaires with incomplete responses, 351 valid questionnaires were obtained, with a valid collection rate of 87.75%. Among them, 42.2% (148) were male and 57.8% (203) were female; 65.0% (228) were aged 70 or above, followed by 19.7% (69) aged 60–69; 51.6% (181) had education levels below elementary school, followed by 28.2% (99) who could not read or write. 43.9% (154) had their spouses as main caregivers, followed by 36.5% (128) who had children as their main caregivers.

### 3.3. Data Analysis

The data analysis of this study was divided into two parts. The first part was a descriptive statistical analysis and a reliability analysis using SPSS 28.0 statistical package. Cronbach’s alpha coefficient was used to conduct the reliability analysis. Cronbach’s alpha is a measure of the internal consistency of a scale. Internal consistency refers to the extent to which all measurement items in a scale measure the same construct [66]. The second part was to validate the research model by using AMOS 28.0 software for statistical methods such as confirmatory factor analysis and structural equation modelling. Confirmatory factor analysis is a statistical technique which is used to verify the factor structure of a set of observed variables. Structural equation modelling is to verify complex structural relationships between one or more measured variables and latent constructs [67]. In the data analysis, a two-step approach proposed by Anderson and Gerbing [68] was used in this study. In the first step, a measurement model was used to assess the reliability and validity, and in the second step, a structural model was used for an overall assessment.

## 4. Results

### 4.1. Common Method Variance (CMV)

Since this study adopted a single self-reported questionnaire to collect cognitive information on all constructs from community elders, there may be a concern of common method variance (CMV) in the research methodology. This is because the self-reported scale was administered to a single source of subjects at the same point in time, and the correlation between the constructs was inflated due to the generalization about assessments, resulting in single-source bias [69]. Harman’s single-factor test was used in this study to detect CMV. All questionnaire items were conducted to exploratory factor analysis without rotation, and the results showed that a total of six factors were extracted, and the explanatory power of the first factor was 38.392%, which did not reach 50%, as shown in Table 1, so there should not be a serious effect of single-source bias. 

### 4.2. Measurement Model

The study measured the reliability of the questions in terms of item reliability and construct reliability, where the item reliability was measured by the factor loadings and the squared multiple correlations (SMC). The results showed that the factor loadings of each questionnaire item were greater than the threshold of 0.5 and the SMC values of each item were greater than the threshold of 0.2, as shown in Table 2. The results indicated that the Cronbach’s α values of the scales ranged from 0.855 to 0.974, which exceeded the threshold of 0.7 suggested by Nunnally [70]. Taken together, this indicates that the internal consistency of the constructs is good.

In validity analysis, the study adopted confirmatory factor analysis to measure the convergent validity, and according to Hair et al. [71], used composite reliability (CR) and average variance extracted (AVE) to measure the convergent validity of each construct. The CR measures the overall reliability of all the observed variables for each potential construct, while the AVE measures the average explanatory variance of the observed variables for each latent construct. As shown in Table 2, the CR values of all the constructs in this study were greater than 0.7, and the AVE values were all greater than 0.5, indicating good convergent validity of each construct. 

Discriminant validity is a measure of the degree of difference between each construct with its observed variables and other constructs with their observed variables. According to the criteria suggested by Fornell and Larcker [72], the correlation coefficient between two constructs should be less than the square root of the AVE of each construct to indicate that the construct has discriminant validity. As shown in Table 3, the correlation coefficients between all the constructs and any other constructs were smaller than the square root of the AVE of each construct, which indicated that the constructs had good discriminant validity.

### 4.3. Structural Model

In this step, a structural model analysis was conducted. This study determined the goodness of fit of the hypothetical model to the actual data using the goodness of fit statistics proposed by previous studies to assess how the overall model fits the observed data. In terms of the overall goodness of fit of the structural equation model, according to the suggested threshold values for each goodness of fit metric by Gefen et al. [73] and Gefen et al. [74], the model goodness of fit of this study was χ^2^/df = 2.314 (recommended value < 3), GFI = 0.905 (recommended value > 0.90), AGFI = 0.867 (recommended value > 0.80), NFI = 0.943 (recommended value > 0.90), CFI = 0.966 (recommended value > 0.90), IFI = 0.967 (recommended value > 0.90), RMR = 0.030 (recommended value < 0.050), RMSEA = 0.061 (recommended value < 0.080). All of the above indicators are within the suggested thresholds, which indicates satisfactory overall goodness of fit of the theoretical model.

**Figure 2:**
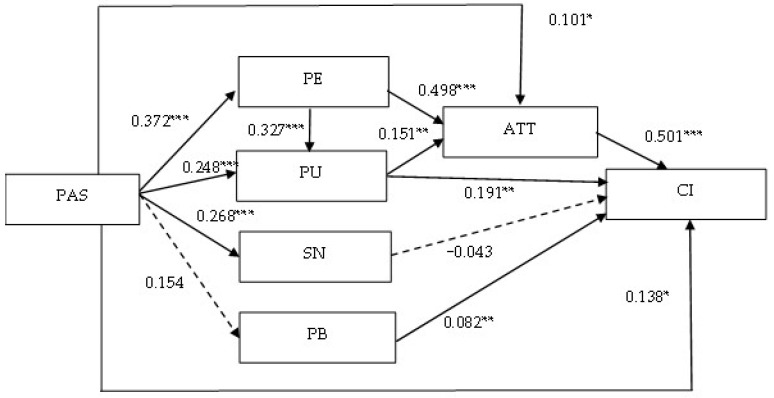
The Final Model and Path Coefficients. Note: * Significant at 0.05 level. ** Significant at 0.01 level. *** Significant at 0.001 level.

To verify the hypothetical relationships, as shown in Table 4 and Figure 2, we present the hypotheses description, path coefficients, and test results in a table. First, the continuance intention to use telehealth was directly and significantly influenced by attitude, perceived behavioral control, perceived usefulness, and perceived autonomy support. Therefore, hypotheses H1, H3, H4, and H8 are supported. Second, users’ attitudes toward telehealth were directly and significantly predicted by perceived usefulness, perceived ease of use, and perceived autonomy support jointly. Therefore, hypotheses H5, H6, and H9 are supported. Furthermore, users’ perceived ease of use of telehealth was directly and significantly predicted by perceived autonomy support. Therefore, hypothesis H12 is supported. Users’ perceived usefulness of telehealth is directly and significantly influenced by perceived ease of use and perceived autonomy support. Therefore, hypotheses H7 and H13 are supported. Finally, users’ subjective norms for telehealth are directly and significantly influenced by perceived autonomy support. Therefore, hypothesis H10 is supported. Among the hypotheses proposed in this study, H2 (subjective norm positively influences continuance intention) and H11 (perceived autonomy support positively influences perceived behavioral control) were unsupported. 

Table 5 lists the direct, indirect and total effects in the research model. As shown in Table 5, attitude exerted the strongest total effect on continuance intention, followed by perceived autonomy support, perceived ease of use, perceived usefulness, perceived behavioral control, and subjective norm. 

## 5. Discussion

### 5.1. Conclusions

The spread of the COVID-19 pandemic has severely limited in-person health care, making the barriers to health care access more acute than ever. Many countries have adopted telehealth in large numbers in order to lower the risk of infection, leading to increased interest and attention in this innovative care technology. The purpose of this study is to develop and validate a causal framework for telehealth by integrating TPB, TAM, and SDT. The findings reveal that: (1) Perceived autonomy support directly and significantly influences perceived ease of use, perceived usefulness, subjective norm, attitude, and continuance intention. (2) Perceived usefulness is directly and significantly influenced by perceived ease of use and perceived autonomy support. (3) Attitude is directly and significantly influenced by perceived usefulness, perceived ease of use, and perceived autonomy support. (4) Continuance intention is directly and significantly influenced by attitude, perceived behavioral control, perceived usefulness, and perceived autonomy support. (5) In terms of direct effect, attitude is the most influential factor on continuance intention, followed by perceived autonomy support, perceived usefulness, perceived behavioral control, and subjective norm. (6) In terms of total effect, attitude is the most influential factor on continuance intention, followed by perceived usefulness, perceived autonomy support, perceived behavioral control, and subjective norm. (7) In terms of mediating effect, perceived ease of use, perceived usefulness, and attitude have significant mediating effects.

An important social contextual factor in SDT, perceived autonomy support, plays an important role as an antecedent variable in the framework of this study and has a direct and significant effect on all variables other than perceived behavioral control. In particular, perceived autonomy support also has significant direct and total effects on continuance intention. This result is similar to Assadi & Hassanein [43]. It shows the importance of perceived autonomy support in residents’ continuance intention of telehealth. The findings support the central thesis of SDT: a social environment with perceived autonomy support helps people maintain intrinsic motivation and internalize extrinsic motivation. That is to say, an authoritative or influential person can accept and acknowledge the internal frame of reference of others, respect others, encourage exploration and choice, support others’ decisions, and avoid coercing or controlling people. Establishing an environment of perceived autonomy support helps facilitate the intrinsic and extrinsic motivations—perceived ease of use, perceived usefulness, and subjective norms, which in turn can influence attitudes and continued adoption of telehealth to shape health behaviors. The role of residents/patients will then be changed from the passive receivers of health care to active participants in the processes of delivering telehealth services [43]. Among the hypotheses that perceived autonomy support influences other variables, the only hypothesis that is not supported is H11 (perceived autonomy support positively influences perceived behavioral control). The reason why the H11 is not supported is inferred to be that the medical institutions that implement telehealth have case managers or volunteers on site to assist the elderly in the community with physiological measurements, eliminating difficulties to use such services for the elderly, and therefore the perceived behavior control is unaffected by perceived autonomy support.

All of the hypotheses regarding TAM in this study are supported, indicating that TAM is applicable in the context of telehealth. The findings are consistent with the findings of Kohnke et al. [35], Tsai [36], Bettiga et al. [6], and Peixoto et al. [3]. Additionally, all hypotheses about TPB are supported except for H2 (subjective norm positively influences continuance intention). The reason why H2 is not supported is inferred to be that the communities tested are mostly in rural areas of Nantou County, where the main caregivers of the elders are mostly their spouses, and most of their children have moved to the cities. As a result, there is a general lack of medical knowledge and health awareness, as well as low accessibility of medical care. There is a lack of significant others to remind and urge the elderly to use telehealth services, making it difficult to develop behaviors of regularly monitoring health status, and therefore significant others cannot influence continuance intention.

This study combines three different theories that have been adopted by scholars to explain and predict the behavioral models of telehealth care, namely, SDT, TAM, and TPB, and attempts to build an integrated model and perspective, so that the three theories can complement each other and establish a research framework and orientation for future scholars to explore the behavioral models of telehealth. Overall, the research framework is well validated and can be used as a basis for subsequent research.

### 5.2. Implications

First, in terms of social context factors, perceived autonomy support not only has a direct impact on technical factors (perceived ease of use and perceived usefulness) as well as on social influence factor (subjective norm), but also has direct and indirect impacts on attitudes and continuance intention. Therefore, it is important for health care institutions, health authorities or community care sites that promote telehealth care to create a community environment and climate that is supportive of autonomy. For health care workers, communication and interaction with elders in the community should be supportive rather than controlling. This means respecting and listening to the elders’ thoughts, providing information and education on the health promotion aspects of telehealth care, and explaining the cause-and-effect relationship between telehealth and health management so that the elders can make their own choices about good health behaviors, rather than being forced. In this way, healthy behaviors are more likely to be sustainable.

In recent years, the issue of the physician–patient relationship has received widespread attention from the public. The shared decision making (SDM) is a recent innovative perspective in the healthcare field. SDM refers to a partnership between health care providers and patients. Healthcare providers and patients make decisions for certain medical conditions or possibilities based on mutual consent and taking into account patient preferences. SDM shares a similar perspective with perceived autonomy support in that the health care providers provide complete information to the patients or elderly, listen to the patients’ needs, respect their opinions, and autonomously choose courses of action to achieve better health care quality and outcomes. 

Moreover, for a more autonomy-supportive social context, it is important to build a social network of trust. Telehealth-related education, screening, physical fitness activities or lectures may take place along with the community’s existing religious beliefs, entertainment and leisure, fitness and recreation venues such as temples and churches, parks, activity centers, or community care sites and other places where the elderly gather for their daily activities. This not only helps to build connection between health care providers and the general public, but also strengthens the trust between physicians and patients while bringing them closer. When there is a strong psychological connection between the elderly and health care providers in the community, elders’ participation in telehealth can be better promoted, and the relevant health promotion programs can be better implemented. 

In terms of technological factors, perceived ease of use and perceived usefulness have an important mediating effect between autonomy support and attitudes. The quality of telehealth technology is highly relevant to the attitudes of elders in the community. Since many elderly people’s physiological functions such as vision, hearing, and limbs may have deteriorated or become disabled due to aging, the design of hardware devices and software functions must take into account such special physiological factors. Also, the data of physiological measurements should be accurate and reliable to be trusted by the elderly and their families, as well as to allow the healthcare professionals to carry out customized health management, medical diagnosis, and health promotion programs for the elderly. Organizations implementing telehealth care should strengthen the IT service quality to provide timely, reliable, trustworthy, and warm services in response to community members’ feedback on the system, which will in turn have a better effect on the elderly’s attitude toward using the technology.

Although subjective norm does not have a significant effect on the continuance intention, Kuo et al. [27] shows that subjective norms have a significant effect on the behavioral intentions of experienced physicians with telemedicine. Therefore, future implementers should continue to strengthen the community’s role in long-term care by providing specific incentives or benefits to encourage people to participate in community-based activities. For example, the providers may offer prizes for 100 telehealth visits. The providers may also send alert messages of physiological measurement abnormality to the elderly’s family members or important relatives via text message or mobile apps, provide contact information of medical personnel, and visit the elderly in person where appropriate, in order to enhance continuance intention.

Finally, the study reveals that perceived behavioral control is a significant predictor of continuance intention. Organizations providing telehealth should consistently provide community residents with adequate software and hardware, including physiological measurement devices, health data platforms, and quality professional service teams, such as case managers and volunteers, so that the elderly are less likely to be afraid of or reject new technologies, and their ability to operate the system can improve, thereby enhancing continuance intention. 

In this current study, we integrate the theory of planned behavior (TPB), technology acceptance model (TAM), and self-determination theory (SDT) to understand the adoption behavior of a telehealth system. Healthcare organizations and professionals should keep in mind the crucial factors of continuance intention of residents when introducing and implementing innovative technologies in healthcare context like telehealth systems. These influencing factors include autonomy-supportive social context, user-friendly and useful system, as well as a trusting climate of community, and supporting resources. Once these antecedents are more improved, the residents will play more active roles in their health and welfare, which in turn, will promote the vision of healthy aging in the community to be accomplished. As such, the multi-perspective framework of this study should provide innovative and valuable contributions to practitioners and scholars.

### 5.3. Limitations and Future Research

Since the sample for this study is drawn from people who have used community-based telehealth services in Nantou County, there may be limitations in generalizing the results of this study to other countries or cities. Future studies could be conducted in a wider range of areas, such as metropolitan areas, and differential analysis could be conducted to provide more valuable insights. Also, possible directions of future research may include application and extension of our research framework in specific diseases or other smart health technologies (such as m-health, telemedicine, personal health record, artificial intelligent). To evaluate the performance of telehealth, health impact and satisfaction assessment of the research is also needed [43]. This will be important in guiding the development of creative smart health technologies [75].

Due to the fact that this study adopts a cross-sectional research approach, measurement of the subjects’ cognition and feelings is limited to the time of testing, and it was not possible to deeply understand the context of personal attitudes or emotional changes of the sampled subjects. Therefore, future studies may also be conducted with a longitudinal approach, where in-depth observation of various research concepts in framework at different time points should lead to more insights.

The COVID-19 pandemic has created a focus on telehealth, and related intelligent products, such as wearable devices and intelligent sensors, have been widely used in medical and long-term care settings, making intelligent health technology one of the most-discussed health-related topics today. It is recommended that future studies should adjust and extend this research to different countries and cultures to provide theoretical and practical breadth and depth, and to enhance the health and well-being of senior citizens.

## Figures and Tables

**Table 1:** Harman’s Single-Factor Test Results.

Factor	Eigenvalue	Proportion of Explained Variance (%)	Cumulative Proportion of Explained Variance (%)
1	8.062	38.392	38.392
2	2.689	12.806	51.198
3	2.196	10.459	61.657
4	1.419	6.757	68.414
5	1.208	5.752	74.166
6	1.108	5.278	79.443

**Table 2:** Measurement Model Results.

Construct	Mean	SD	Cronbach’s α	CR	AVE
Perceived Autonomy Support	4.199	0.461	0.919	0.905	0.620
Perceived Ease of Use	4.158	0.534	0.969	0.970	0.915
Perceived Usefulness	4.165	0.497	0.974	0.805	0.673
Subjective Norm	3.822	0.635	0.957	0.957	0.918
Perceived Behavior Control	3.742	0.872	0.855	0.858	0.752
Attitude	4.273	0.479	0.911	0.912	0.720
Continuance intention	4.303	0.537	0.938	0.939	0.885

**Table 3:** Discriminant Validity Analysis Results.

	1	2	3	4	5	6	7
1. Perceived Autonomy Support	(0.787)						
2. Perceived Ease of Use	0.357 ***	(0.957)					
3. Perceived Usefulness	0.392 ***	0.422 ***	(0.820)				
4. Subjective Norm	0.194 ***	0.229 ***	0.230 ***	(0.958)			
5. Perceived Behavior Control	0.067	0.158 **	0.203 ***	0.273 ***	(0.867)		
6. Attitude	0.413 ***	0.638 ***	0.411 ***	0.218 ***	0.076	(0.849)	
7. Continuance intention	0.393 ***	0.485 ***	0.415 ***	0.123 *	0.146 *	0.511 ***	(0.941)

Note: The diagonal elements of the matrix are the square root of the average variance extracted (AVE). The inter-construct correlations are shown off the diagonal. * Significant at 0.05 level. ** Significant at 0.01 level. *** Significant at 0.001 level.

**Table 4:** Research Hypotheses Test Results.

Hypothesis	Path Coefficient	Supported
H1: Attitude → Continuance Intention	0.501 ***	Yes
H2: Subjective Norm → Continuance Intention	−0.043	No
H3: Perceived Behavioral Control → Continuance Intention	0.082 **	Yes
H4: Perceived Usefulness → Continuance Intention	0.191 **	Yes
H5: Perceived Usefulness → Attitude	0.151 **	Yes
H6: Perceived Ease of Use → Attitude	0.498 ***	Yes
H7: Perceived Ease of Use → Perceived Usefulness	0.327 ***	Yes
H8: Perceived Autonomy Support → Continuance Intention	0.138 *	Yes
H9: Perceived Autonomy Support → Attitude	0.101 *	Yes
H10: Perceived Autonomy Support → Subjective Norm	0.268 ***	Yes
H11: Perceived Autonomy Support → Perceived Behavioral Control	0.154	No
H12: Perceived Autonomy Support → Perceived Ease of Use	0.372 ***	Yes
H13: Perceived Autonomy Support → Perceived Usefulness	0.248 ***	Yes

* Significant at 0.05 level. ** Significant at 0.01 level. *** Significant at 0.001 level.

**Table 5:** Direct, Indirect and Total Effects Analysis Results.

Construct	Direct Effect	Indirect Effect	Total Effect	Rank of Total Effect
Perceived autonomy support	0.138	0.242	0.380	2
perceived ease of use	-	0.336	0.336	3
perceived usefulness	0.191	0.076	0.267	4
subjective norm	−0.043	-	−0.043	6
perceived behavioral control	0.082	-	0.082	5
attitude	0.501	-	0.501	1

## Data Availability

Data generated during the study. The data presented in this study are available on request from the corresponding author.
